# Overexpression of hepatic serum amyloid A1 in mice increases IL-17-producing innate immune cells and decreases bone density

**DOI:** 10.1016/j.jbc.2021.100595

**Published:** 2021-03-26

**Authors:** Minjee Choi, Song Park, Jun Koo Yi, Wookbong Kwon, Soyoung Jang, Si-Yong Kim, Wookyung Yu, Myoung Ok Kim, Zae Young Ryoo, Seong-Kyoon Choi

**Affiliations:** 1 Core Protein Resources Center, DGIST, Daegu, Republic of Korea; 2 Department of Brain and Cognitive Sciences, DGIST, Daegu, Republic of Korea; 3 Gyeongsangbukdo Livestock Research Institute, Yeongju-si, Republic of Korea; 4 Division of Biotechnology, DGIST, Daegu, Republic of Korea; 5 School of Life Science, BK21 FOUR KNU Creative Bioresearch Group, Kyungpook National University, Daegu, Republic of Korea; 6 School of Animal Science Biotechnology, Kyungpook National University, Sangju-si, Republic of Korea

**Keywords:** interleukin 17A, osteoclasts, osteoblasts, innate immunity, bone, transgenic mice, serum amyloid A, G-CSF, granulocyte colony-stimulating factor, RANKL, receptor activator of nuclear factor-κB ligand, SAA, serum amyloid A, TG, transgenic mice

## Abstract

Serum amyloid A (SAA) is an acute-phase protein produced primarily in the liver that plays a key role in both the initiation and maintenance of inflammation. Rapidly secreted SAA induces neutrophilia at inflammatory sites, initiating inflammation and inducing the secretion of various cytokines, including TNF-α, IL-6, and IL-17. IL-17 is expressed in several inflammatory cells, including innate immune cells such as γδT cells, ILC3 cells, and neutrophils. Increased IL-17 levels exacerbate various inflammatory diseases. Among other roles, IL-17 induces bone loss by increasing receptor activator of nuclear factor-κB ligand (RANKL) secretion, which stimulates osteoclast differentiation. Several studies have demonstrated that chronic inflammation induces bone loss, suggesting a role for SAA in bone health. To test this possibility, we observed an increase in IL-17-producing innate immune cells, neutrophils, and γδT cells in these mice. In 6-month-old animals, we detected increased osteoclast-related gene expression and IL-17 expression in bone lysates. We also observed an increase in neutrophils that secreted RANKL in the bone marrow of TG mice. Finally, we demonstrated decreased bone mineral density in these transgenic (TG) mice. Our results revealed that the TG mice have increased populations of IL-17-producing innate immune cells, γδT cells, and neutrophils in TG mice. We additionally detected increased RANKL and IL-17 expression in the bone marrow of 6-month-old TG mice. Furthermore, we confirmed significant increases in RANKL-expressing neutrophils in TG mice and decreased bone mineral density. Our results provide evidence that chronic inflammation induced by SAA1 causes bone loss *via* IL-17-secreting innate immune cells.

Serum amyloid A (SAA) is a major acute-phase response protein produced primarily in the liver. Under acute-phase conditions, serum SAA levels can increase by up to 1000 fold. Secreted SAA also induces neutrophilia *via* increased granulocyte colony-stimulating factor (G-CSF) secretion (1). SAA-induced neutrophilia also initiates inflammation and increases immune responses *via* increased secretion of several inflammatory cytokines, including TNF-α, IL-6, IL-1β, and IL-17 (1, 2, 3, 4, 5). Consequently, increased SAA is associated with a variety of inflammatory diseases, including psoriasis (6, 7), rheumatoid arthritis (8), and obesity (9).

Of the cytokines secreted by SAA signaling, IL-17 plays an important role in increasing inflammation by activating neutrophils and attracting inflammatory cells to sites of inflammation (10). Thus, increased IL-17 expression is associated with the exacerbation of inflammatory disease (11). Various innate immune cells, including γδT cells, group 3 innate lymphoid cells (ILC3), and neutrophils are also known to secrete IL-17 (10).

Bone is maintained *via* a balance of osteoclast and osteoblast cell populations, the functions of which are to degrade old bone and generate new bone, respectively (12). Receptor activator of nuclear factor-κB ligand (RANKL) is a signaling molecule that promotes osteoclast differentiation by binding to its receptor RANK, which is expressed in osteoclast precursors (13). IL-17 secretion increases the proportion of osteoclasts by increasing RANKL secretion from osteoblasts. Also, IL-17-producing cells, including Th17 cells and γδT cells, express RANKL themselves (14, 15). Moreover, several studies have reported that neutrophils can also secrete RANKL (16, 17); therefore, IL-17 alters bone homeostasis in a direction that induces bone loss. Also increased IL-17-mediated bone loss has been reported in several chronic inflammatory diseases, including psoriasis (18, 19).

In this study, we sought to determine whether increased SAA induced bone loss *via* increased IL-17 signaling, using SAA1-overexpressing transgenic (TG) mice. We observed an increase in IL-17-producing innate immune cells, neutrophils, and γδT cells in these mice. In 6-month-old animals, we detected increased osteoclast-related gene expression and IL-17 expression in bone lysates. We also observed an increase in neutrophils that secreted RANKL in the bone marrow of TG mice. Finally, we demonstrated decreased bone mineral density in these TG mice. Therefore, we concluded that SAA induced bone loss upon chronic inflammation *via* IL-17-production in innate immune cells. The SAA1 TG mouse model may have additional value as a disease model for IL-17-mediated bone loss.

## Results

### SAA1-overexpressing TG mice display increased IL-17-expressing innate immune cells

**Figure 1:**
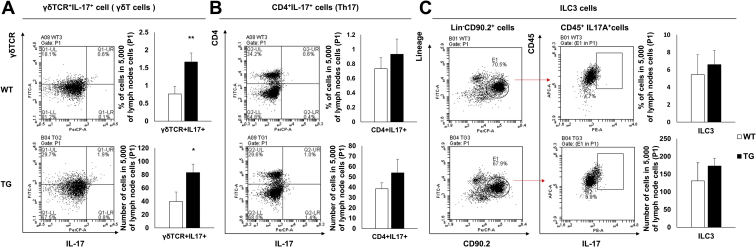
**Increased IL-17-expressing γδT cells in SAA1 TG mice.***A*, *left*, flow cytometry detection of lymph node IL-17A^+^ γδ T cell receptor (TCR)^+^ cells, (*B*, *left*) IL-17A^+^ CD4^+^ (Th17) cells, and (*C*, *left*) lineage^−^CD45^+^CD90.2^+^IL-17A^+^ (ILC3) cells, represented by dot blots. Data are representative of quadruplicate experiments. (*A*–*C*, *right*, *upper*) The percentages of IL-17A^+^ γδ TCR^+^, Th17, and ILC3 cells in 5000 lymph node cells (P1) are shown (n = 4). (*A*–*C*, *right*, *bottom*) The absolute numbers of IL-17A^+^ γδ TCR^+^, Th17, and ILC3 cells per 5000 lymph node cells (P1) are shown (n = 4). Detailed gating strategies and information for P1 are shown (Fig. S1). Data are expressed as the mean ± standard deviation, and unpaired two-tailed Student's *t*-test was applied to compare two groups. ∗*p* < 0.05 and ∗∗*p* < 0.01 compared with WT mice.

In a previous study, we observed that SAA1 increased IL-17 expression *via* the Toll-like receptor 2 (TLR2) in γδT cells (20). In this study, we sought to determine whether IL-17 was secreted by other immune cells, in addition to γδT cells in TG mice. Using flow cytometry, we analyzed the population of IL-17^+^γδTCR^+^ cells (IL-17-producing γδT cells, Fig. 1*A*), IL-17^+^CD4^+^ cells (IL-17-producing Th17 cells, Fig. 1*B*), and lineage^−^CD45^+^CD90.2^+^IL-17A^+^ cells (IL-17-producing ILC3, Fig. 1*C*) in lymph nodes. As observed, IL17^+^ γδTCR^+^ cells were significantly increased in TG mice when compared with WT mice. However, the proportion of Th17 and ILC3 cells did not differ significantly between these mice. Therefore, we confirmed that SAA1 overexpression in mice led to an increased γδT cell population, but not Th17 or ILC3 cells.

### SAA1-overexpressing TG mice display increased neutrophil populations

**Figure 2:**
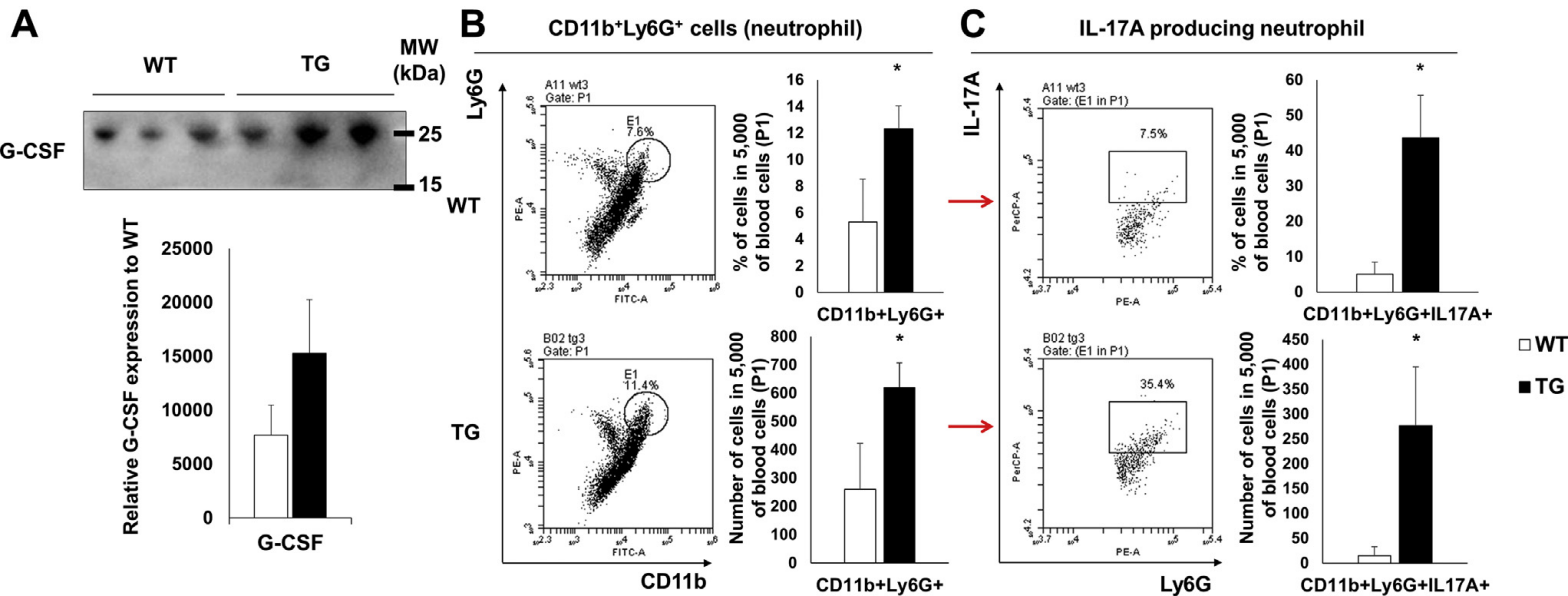
**Increased IL-17-expressing neutrophils in SAA1 TG mice.***A*, *upper*, circulating G-CSF levels measured by western blot using equal volumes (1 μl) of serum. The molecular weight of G-CSF is 19 kDa (*A*, *bottom*). The ponceau S staining to show that the same volume of serum was loaded is shown (Fig. S2). Densitometry analysis of western blots showing G-CSF quantities, n = 3. *B*, *left*, blood CD11b^+^Ly6G^+^ cells (neutrophils) detected by flow cytometry are represented by a dot blot, n = 4. *B*, *right*, the percentages of CD11b^+^Ly6G^+^ cells and absolute numbers of CD11b^+^Ly6G^+^ cells per 5000 blood cells (P1) are shown (n = 4). *C*, *left*, CD11b^+^Ly6G^+^ cells gated to IL-17A^+^Ly6G^+^ cells (IL-17-producing neutrophils) by flow cytometry are represented by a dot blot (n = 4). *C*, *right*, the percentage of IL-17A^+^CD11b^+^ Ly6G^+^ cells and absolute numbers of cells per 5000 blood cells (P1) are shown (n = 4). Detailed gating strategies and information for P1 are shown (Fig. S3). Data are expressed as the mean ± standard deviation, and unpaired two-tailed Student's *t*-test was applied to compare two groups. ∗*p* < 0.05 compared with WT mice. The *red arrow* indicates that *A* is gated to *B*. MW, molecular weight.

It was previously reported that SAA increased neutrophilia by increasing G-CSF (1). Neutrophils themselves can express IL-17 (10). To confirm this in our TG mice, we measured G-CSF expression by western blot of serum proteins. We observed increased G-CSF expression in TG mice compared with WT mice (Fig. 2*A*). We next measured neutrophil population *via* flow cytometry. We confirmed the presence of a circulating CD11b^+^ Ly6G^+^ population (neutrophils) in blood, and this was significantly increased in TG mice when compared with WT mice (Fig. 2*B*). In addition, neutrophils in TG mice were found to secrete IL-17 (Fig. 2*C*). Consequently, SAA1 TG mice were confirmed as secreting IL-17 from neutrophils and γδT cells.

### Increased osteoclast-related mRNA expression with increased IL-23 and IL-17 in SAA1-overexpressing TG mice

**Figure 3:**
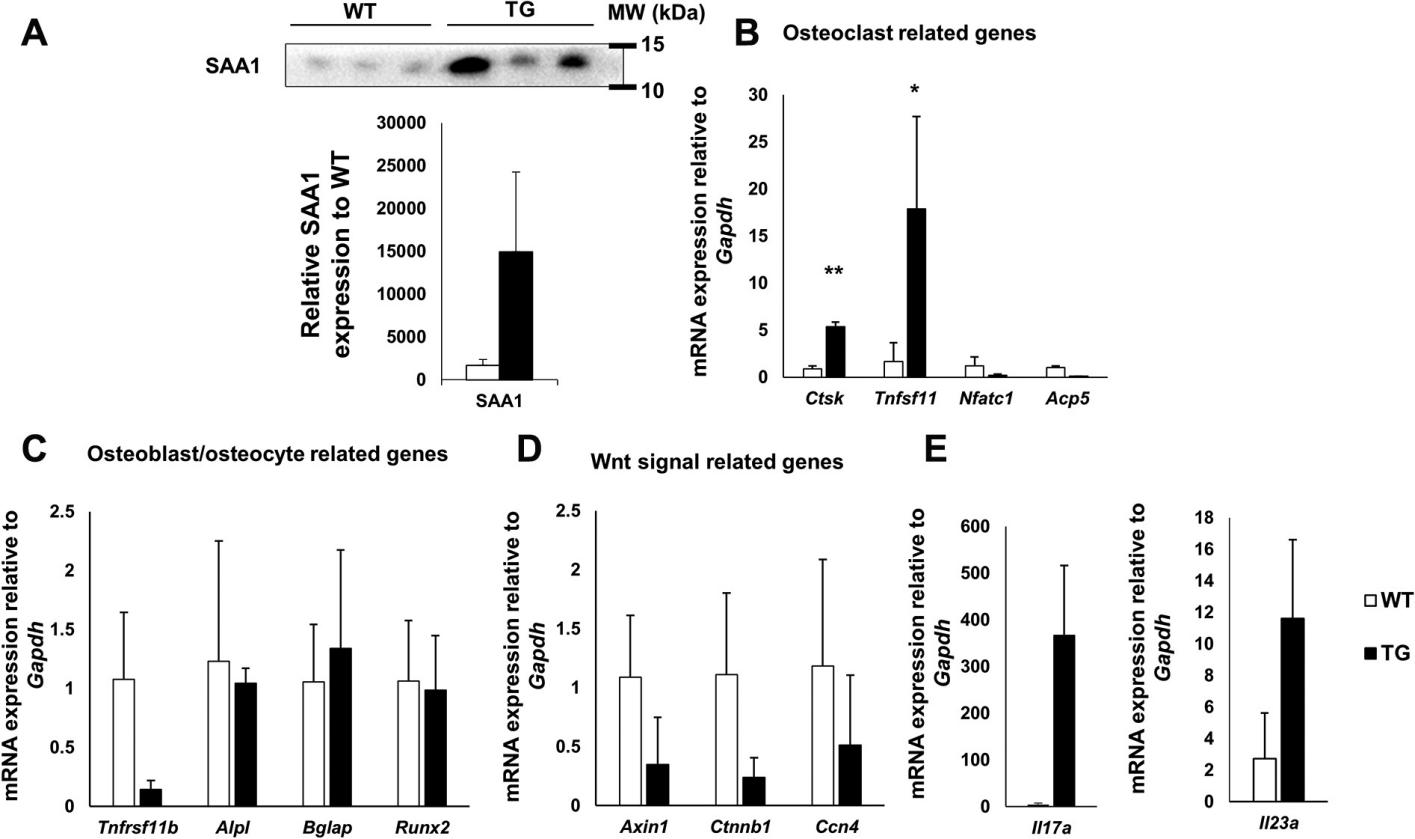
**Increased osteoclast-related gene expression and IL-17 and IL-23 expression in whole bone lysates from 6-month-old SAA1-overexpressing TG mice.***A*, *upper*, circulating SAA1 levels measured by western blot using equal volumes (1 μl) of serum from 6-month-old mice. The molecular weight of SAA1 is 12 kDa (*A*, *bottom*). Densitometry analysis of western blots showing SAA1 quantities, n = 3. The ponceau S staining to show that the same volume of serum was loaded is shown (Fig. S2). Relative mRNA expression levels of (*B*) *Ctsk*, *Tnfsf11*, *Nfatc1*, and *Acp5* (*C*) *Tnfrsf11b*, *Alpl*, *Bglap*, and *Runx2* (*D*) *Axin1*, *Ctnnb1*, and *Ccn4*, and (*E*) *Il17a* and *Il23a*, normalized to glyceraldehyde-3-phosphate dehydrogenase (*Gapdh*) mRNA expression levels, and relative to WT mice in whole bone lysates. For *B–E*; WT; n = 4 and TG; n = 8. Data are expressed as the mean ± standard deviation, and unpaired two-tailed Student's *t*-test was applied to compare two groups. ∗*p* < 0.05 and ∗∗*p* < 0.01 compared with WT mice. MW; molecular weight.

As other studies have reported bone loss mediated by chronic inflammation (18, 19), we also investigated whether bone loss occurred as a result of chronic inflammation induced by SAA1 and IL-17. First, we measured increased SAA1 expression in the serum of 6-month-old mice by western blot. We confirmed increased SAA1 secretion in 6-month-old TG mice compared with WT mice (Fig. 3*A*). We investigated the expression of the following genes: those related to osteoclast activity; *Ctsk*, *Tnfsf11*, *Nfatc1*, and *Acp5* (21), osteoblast/osteocyte activity; *Tnfrsf11b*, *Alpl*, *Bglap*, and *Runx2* (22), and Wnt signaling related genes required for proper osteoblast function (23, 24); *Axin1 Ctnnb1*, and *Ccn4*. No significant differences were observed in the mRNA expression of osteoclast and osteoblast-related markers in whole bone lysates from 7-week-old mice (data not shown). However, when we examined 6-month-old TG mice to confirm the effects of chronic inflammation on bone from increased SAA1 and IL-17, we observed that the osteoclast-related genes, *Tnfsf11* and *Ctsk* were significantly increased in TG mice when compared with WT mice (Fig. 3*B*). We observed no significant differences in osteoblast-associated gene levels between TG and WT mice; however, for *Axin1* and *Ctnnb1* genes associated with Wnt signaling, and *Tnfrsf11b* associated with osteoblast activity, these levels tended to decrease in TG mice (Fig. 3, *C* and *D*). We measured the expression levels of *Il-17a* and *Il-23a* mRNA in bone lysates and confirmed a 367-fold and 11-fold increase, respectively, in TG mice when compared with WT mice (Fig. 3*E*). Therefore, we concluded that chronic inflammation in 6-month-old TG mice induced an increase in osteoclast-related gene expression in relation to IL-17.

### Increased *Tnfsf11* mRNA expression in the bone marrow from SAA1-overexpressing TG mice

**Figure 4:**
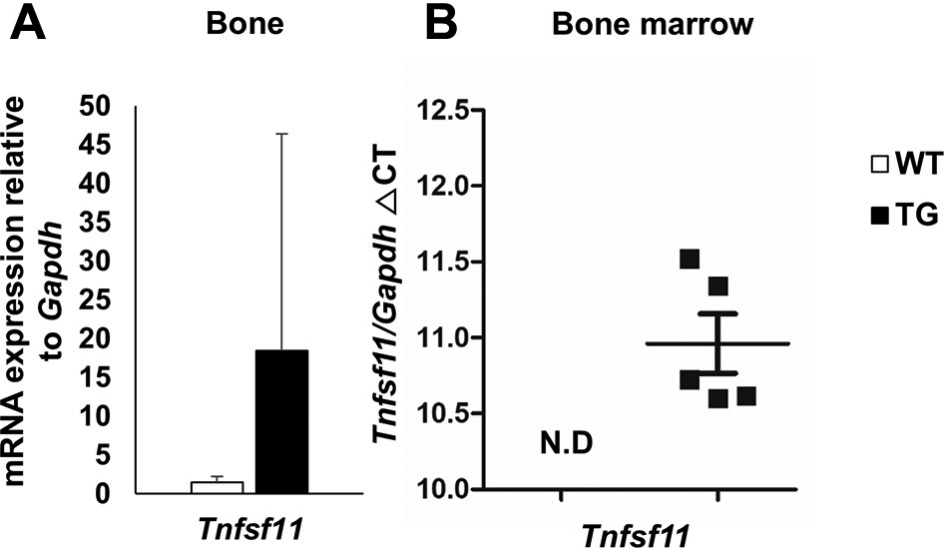
**Increased *Tnfsf11* mRNA expression levels in bone marrow from 6-month-old SAA1-overexpressing TG mice.***A*, relative mRNA expression levels of *Tnfsf11* normalized to glyceraldehyde-3-phosphate dehydrogenase (*Gapdh*) mRNA expression levels and relative to WT mice in bone lysates. *B*, *Tnfsf11/Gapdh ΔCT* was calculated by subtracting the CT value for mRNA *Gapdh* expression levels from the CT value for *Tnfsf11* mRNA expression levels. Data are expressed as the mean ± standard deviation, and unpaired two-tailed Student's *t*-test was applied to compare two groups. For *A* and *B*; WT; n = 4, TG; n = 5. N.D; not detected.

A recent report suggested that osteocytes could also express RANKL (*i.e.*, *Tnfsf11*) when stimulated by IL-17 (15). To investigate RANKL expression in osteocytes, we separated bone and bone marrow. As a result, bone that located osteocyte expressed *Tnfsf11* mRNA in both WT and TG mice, and there was no significant difference (Fig. 4*A*). For bone marrow, *Tnfsf11* mRNA expression was not detected by qPCR in WT mice. However, in TG mice we detected *Tnfsf11* expression (Fig. 4*B*). Therefore, we confirmed that increased *Tnfsf11* expression in TG mice was expressed by bone marrow cells.

### Increased RANKL expression in bone marrow from SAA1-overexpressing TG mice

**Figure 5:**
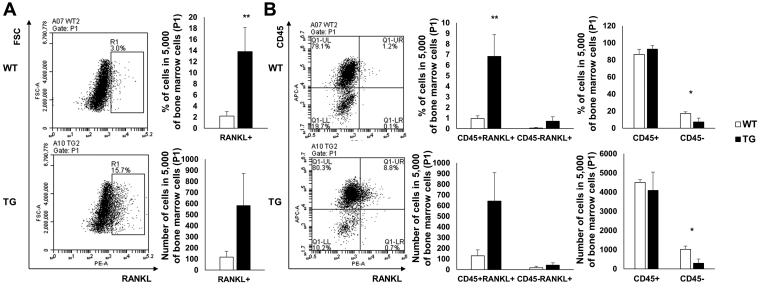
**Increased populations of RANKL-expressing cells in bone marrow from SAA1-overexpressing TG mice.***A*, *left*, Total RANKL^+^ cells and (*B*, *left*) CD45^+^RANKL^+^ and CD45^−^RANKL^+^ cells in bone marrow were detected by flow cytometry and represented by a dot blot (n = 4). Representative data from quadruplicate experiments are shown. The percentage and absolute numbers of (*A*, *right*) RANKL^+^ cells and (*B*, *middle*) CD45^+^RANKL^+^ and CD45^−^RANKL^+^ cells per 5000 of bone marrow cells (P1) are shown (n = 4). *B*, *right*, detailed gating strategies and information for P1 are shown (Fig. S4). Data are expressed as the mean ± standard deviation and unpaired two-tailed Student's *t*-test was applied to compare two groups. ∗*p* < 0.05 and ∗∗*p* < 0.01 compared with WT mice.

We sought to determine whether RANKL protein levels were also increased in TG mice and which bone marrow cells expressed these levels. Firstly, we measured RANKL expression in bone marrow using flow cytometry. Our results indicated that RANKL expression was significantly increased in TG mice when compared with WT mice (Fig. 5*A*). RANKL is known to be expressed by osteoblasts derived from the mesenchymal stem cell lineage (CD45^−^ cells) and inflammatory cells derived from the hematopoietic stem cell (HSC) lineage (CD45^+^ cells). Therefore, we investigated CD45^+^RANKL^+^ cell and CD45^−^RANKL^+^ cell populations to identify the major RANKL-secreting cells between these lineage cells. In TG mice, we observed that RANKL expression increased in CD45^+^ cells, but not in CD45^−^ cells. Therefore, we concluded that increased RANKL expression levels in TG mice were secreted from CD45^+^ inflammatory cells of the HSC lineage, rather than from osteoblasts. Furthermore, we observed that CD45^−^ cells were significantly reduced in TG mice (Fig. 5*B*). These results indicated that RANKL expression was increased in inflammatory cells from SAA1 TG mice.

### Increased RANKL-producing neutrophils in bone marrow from SAA1-overexpressing TG mice

**Figure 6:**
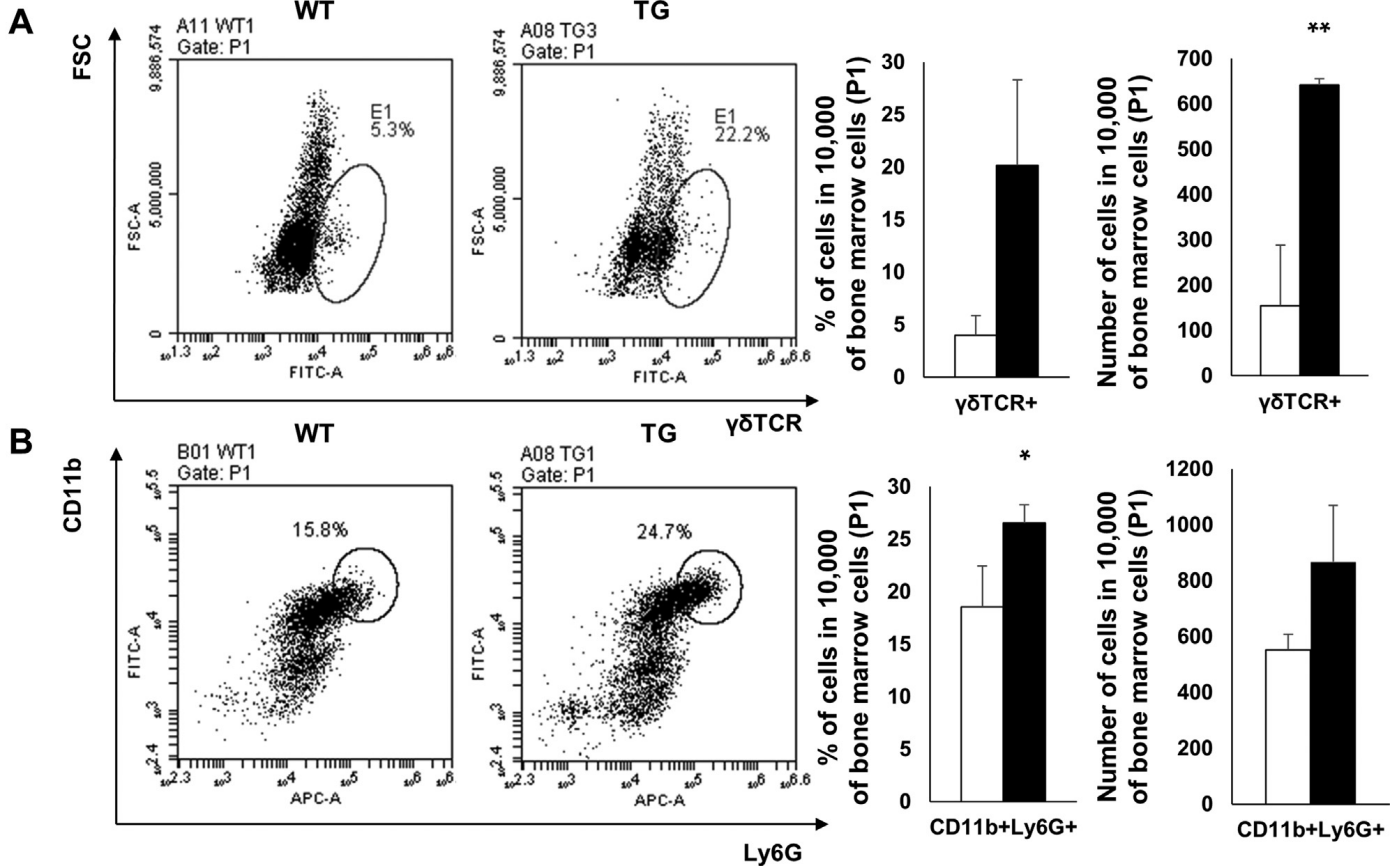
**Increased RANKL-expressing innate immune cells in the bone marrow from 6-month-old SAA1 TG mice.***A*, *left*, γδ TCR^+^ cells and (*B*, *left*) CD11b^+^Ly6G^+^ cells in bone marrow were detected by flow cytometry and represented by *dot blots* (n = 4). Representative data from quadruplicate experiments are shown. The percentages and absolute numbers of (*A*, *right*) γδ TCR^+^ cells and (*B*, *right*) CD11b^+^Ly6G^+^ cells per 10,000 bone marrow cells (P1) are shown. Detailed gating strategies and information for P1 are shown (Fig. S5). Data are expressed as the mean ± standard deviation and unpaired two-tailed Student's *t*-test was applied to compare two groups. ∗*p* < 0.05 and ∗∗*p* < 0.01 compared with WT mice.

**Figure 7:**
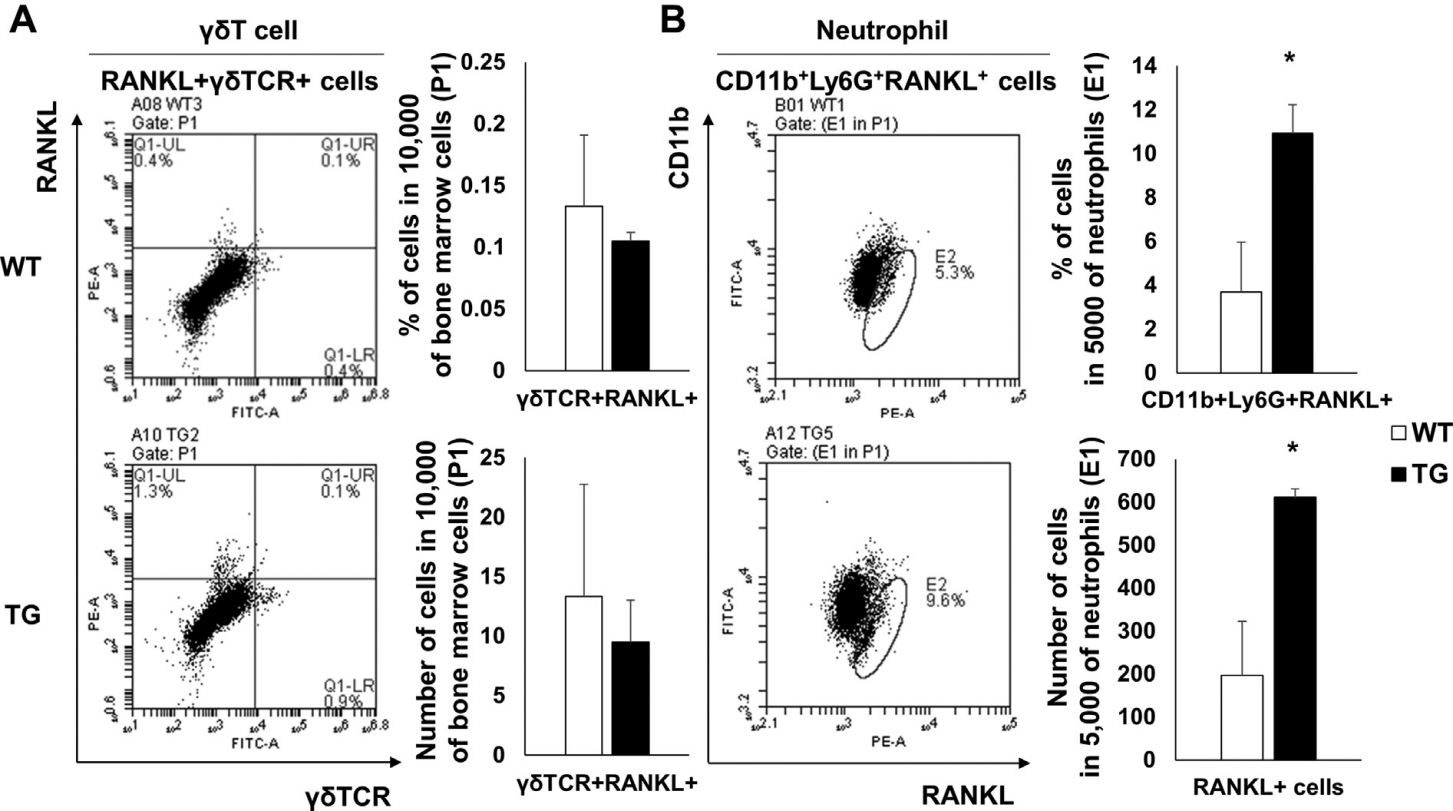
**Increased RANKL-expressing neutrophils in bone marrow from SAA1 TG mice.***A*, *left*, RANKL^+^γδTCR^+^ cells and (*B*, *left*) RANKL^+^CD11b^+^Ly6G^+^ cells in bone marrow were detected by flow cytometry and represented by dot blots (n = 4). Representative data from quadruplicate experiments are shown. *A*, *right*, *upper*, the percentage of RANKL^+^γδTCR^+^ cells and (*A*, *right*, *bottom*), the counts for RANKL^+^γδTCR^+^ cells in 10,000 bone marrow cells (P1) are shown (n = 4). *B*, *right*, *upper*, the percentage of RANKL^+^ cells and (*B*, *right*, *bottom*), the counts for RANKL^+^ cells per 5000 CD11b^+^Ly6G^+^ cells in bone marrow (E1) are shown (n = 4). Detailed gating strategies and information for P1 and E1 are shown (Fig. S6). Data are expressed as the mean ± standard deviation and unpaired two-tailed Student's *t*-test was applied to compare two groups. ∗*p* < 0.05 compared with WT mice.

IL-17-expressing cells themselves are known to be capable of expressing RANKL. Therefore, we ascertained whether IL-17-expressing innate immune cells were increased in TG mice. Bone marrow neutrophil and γδT cell populations were determined by flow cytometry. From these data, we observed that γδT cell and neutrophil populations were significantly increased in TG mice when compared with WT mice (Fig. 6). Therefore, we examined RANKL expression in γδT cells and neutrophils in the bone marrow of TG mice using flow cytometry. We confirmed that γδT cells did not express RANKL (Fig. 7*A*). However, in neutrophils, RANKL expression was significantly increased in TG mice when compared with WT mice (Fig. 7*B*), indicating that IL-17-expressing neutrophils secreted RANKL and contributed to osteoclast differentiation in SAA1 TG mice.

### Reduced bone mineral density and increased TRAP-positive cells in SAA1-overexpressing TG mice

**Figure 8:**
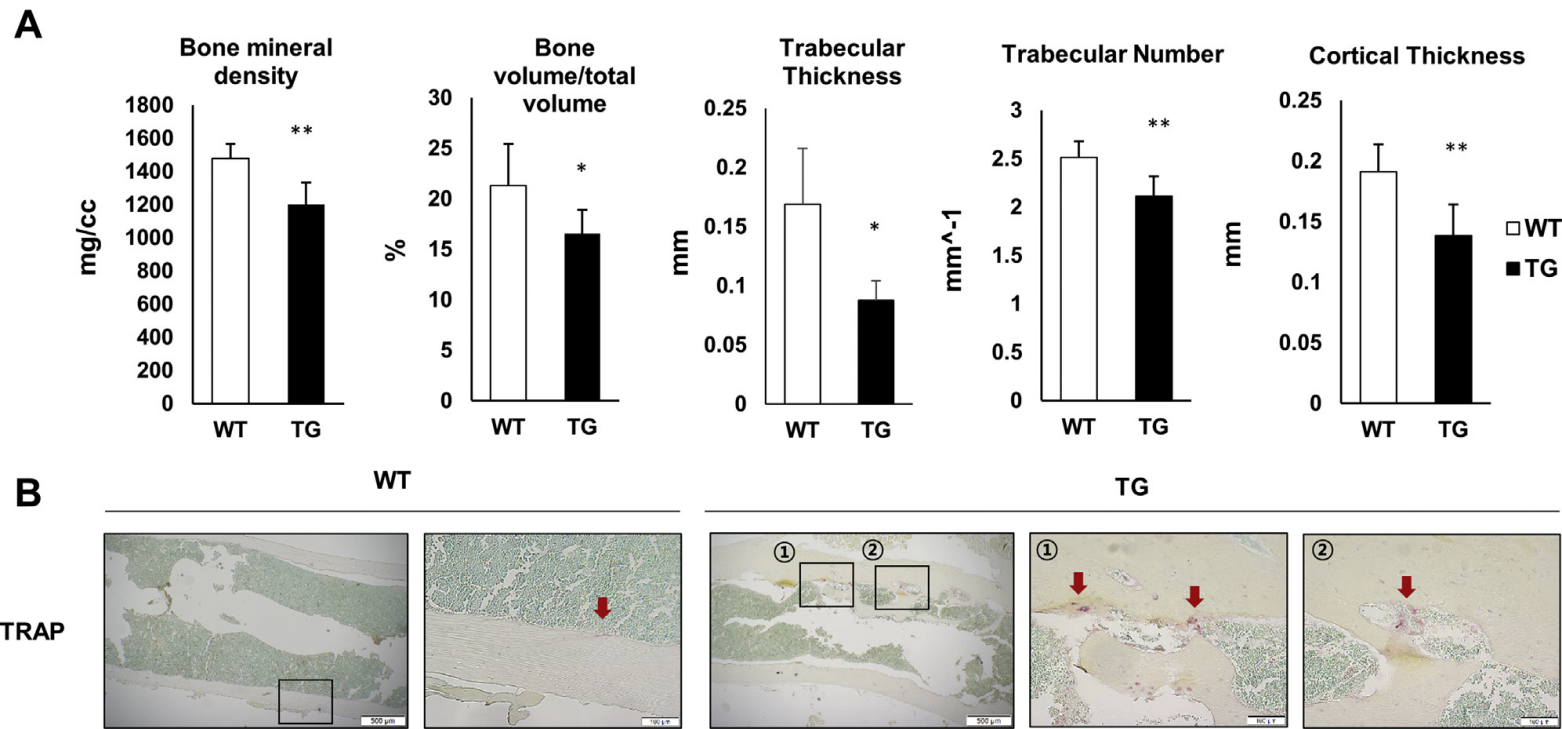
**Changes in bone mineral density and architecture in SAA1 TG mice.***A*, *upper*, bone mineral density, bone volume/total volume, trabecular thickness, trabecular number, and cortical thickness of SAA1 TG and WT mice (n = 8). *B*, a representative TRAP stained section from a quadruplicate experiment is shown. The red arrows indicate TRAP-positive areas (n = 4). (WT, TG, *left*) scale bar = 500 μm, (WT, TG, *middle*, *right*) scale bar = 100 μm. Data are expressed as the mean ± standard deviation and unpaired two-tailed Student's *t*-test was applied to compare two groups. ∗*p* < 0.05 and ∗∗*p* < 0.01 compared with WT mice.

Finally, we investigated whether increased RANKL in SAA1 TG mice resulted in bone loss. Bone mineral density was measured using micro-computed tomography (μCT) in 6-month-old TG mice. We confirmed that bone mineral density, bone volume/total volume, trabecular thickness, trabecular number, and cortical thickness were all significantly decreased in TG mice (Fig. 8*A*). Histological analysis was also performed using TRAP staining in mouse tibia sections; we detected increased TRAP-positive staining in TG mice when compared with WT mice (Fig. 8*B*). Thus, we confirmed that bone mineral density decreases were correlated with increased osteoclasts in TG mice.

## Discussion

It was reported that SAA signaling promotes increased IL-17 expression (4, 5). In our previous study, we demonstrated that SAA1 overexpression in TG mice led to spontaneous psoriasis (20), in which IL-17 plays a key role (25). In this study, we demonstrated that SAA stimulated γδT cells and neutrophils to secrete IL-17. Long-lasting inflammation in several chronic inflammatory diseases, including psoriasis, is related to bone loss (18, 19). Therefore, we investigated whether bone loss occurred in SAA1 TG mice, *via* chronic inflammation with IL-17. We observed that 6-month-old TG mice had decreased bone mineral density and increased RANKL expression, when compared with WT littermate controls. We also observed RANKL-producing neutrophils in these SAA1 TG mice, suggesting that chronic IL-17-secreting neutrophils contributed to decreasing bone mineral density *via* RANKL signaling in SAA1 TG mice.

SAA induces neutrophilia by promoting G-CSF secretion (1, 5). IL-17 accelerates neutrophil infiltration at inflammation sites to both initiate and maintain inflammatory responses (10). Therefore, it is conceivable that interactions between SAA and IL-17 result in neutrophilia in SAA1 TG mice. Despite their short life span, activated neutrophils can produce considerable protein and lipid levels, which participate in inflammatory responses (26, 27). Similarly, neutrophils also produce RANKL (16, 17). It is known that RANKL^+^ neutrophils interact directly with osteoclasts (17). In addition, it was confirmed *in vivo* that RANKL^+^ neutrophils contributed to osteoclastogenesis (28). In this study, we were unable to demonstrate the direct effects of neutrophils on bone formation. However, data from previous studies have allowed us to speculate on the effects of RANKL^+^ neutrophils on bone homeostasis (17, 28). Therefore, our observations that increased numbers of neutrophils, as well as increased IL-17 and RANKL expression levels on surface membranes, suggested that neutrophils played a major role in bone loss in our TG mice.

However, the precise role of IL-17 signaling in osteoclasts and osteoblasts is controversial. IL-17 was recently shown to reduce Wnt signaling when associated with osteoblast function; this decreased activity resulted from a relative increase in osteoclast function (19). Our results demonstrated that *Tnfrsf11b* mRNA expression, which is related to osteoblast function (22), and *Axin1* and *Ctnnb1*, which are related to Wnt signaling (23, 24), were reduced in SAA1 TG mice. In addition, CD45^−^ populations were significantly decreased in TG mice, suggesting that IL-17 may have decreased osteoblast populations *via* Wnt signaling, leading to a relative increase in osteoclast levels. Further studies are required to fully elucidate the effects of IL-17 on osteoblasts and osteoclasts.

In contrast with our data, several studies showed that SAA suppresses osteoclast differentiation (29, 30). The inhibitory effects of SAA in osteoclastogenesis were discovered *in vitro*; however, our experimental data were collected *in vivo*. Also, several studies have shown that IL-17 induces osteoclastogenesis. Therefore, we speculate that the direct effects of SAA on osteoclastogenesis are weaker than any indirect effects of SAA interactions with IL-17 on osteoclast differentiation.

In this study, we revealed that SAA induced bone loss *via* an increase in IL-17-producing innate immune cells. These data suggested that IL-17-mediated inflammatory responses and bone loss may decrease when SAA levels are reduced. Therefore, SAA appears to be a valuable therapeutic target for some diseases characterized by inflammatory responses and bone loss. In addition, we also demonstrated the value of SAA1-overexpressing TG mice as a potential disease model for bone loss caused by chronic IL-17 inflammation.

## Experimental procedures

### Mice

SAA1 TG mice were previously generated in a C57BL/6 background (31). Age-matched (7–9-week-old and 6-month-old) TG mice and littermates (WT mice) were used. Mice were raised and maintained under conventional conditions. They were given *ad libitum* access to food and water and housed under 12 h light/dark conditions. All animal experiments were conducted in accordance with the guidelines for animal experimentation and with permission from the Animal Use and Care Committee of Kyungpook National University.

### Quantitative polymerase chain reaction (q-PCR) analysis

To separate bone marrow from bone, the femur at both ends was severed with sterile scissors. Bone marrow was rinsed using a 23-gage needle and a 10 cc syringe filled with ice-cold phosphate buffered saline. Bone tissue was harvested and ground using a MagNA Lyser (Roche Diagnostics). A TaKaRa Mini-BEST Universal RNA Extraction Kit (TaKaRa) was used to isolate RNA according to manufacturer's instructions. Total RNA was reverse transcribed at 60 °C for 1 h using a PrimeScript first strand cDNA Synthesis Kit (Takara). *Gapdh* was used as an internal control for quantification. Messenger RNA (mRNA) levels were expressed as the fold change in expression when compared with untreated control mice. The primers are shown (Table 1). Expression levels of *Il*-*17a*, *Il*-*23a*, *Acp5*, *Nfatc1*, *Ctsk*, *Runx2*, *Tnfrsf11b*, *Tnfsf11*, *Ctnnb1*, *Axin1*, *Alpl*, *Bglap*, *Ccn4*, and *Gapdh* in samples were determined using SYBR green fluorescence (Takara), using the CFX96 TM Real Time System (Bio-Rad).

**Table 1:** Primers used for quantitative polymerase chain reaction (qPCR)

Gene	Primer sequence
*Il-17a*	Forward: 5′-TCTCCACCGCAATGAAGACC-3′
	Reverse: 5′-CACACCCACCAGCATCTTCT-3′
*Il-23a*	Forward: 5′-GCTGTGCCTAGGAGTAGCAG-3′
	Reverse: 5′-TGGCTGTTGTCCTTGAGTCC-3′
*Ctsk*	Forward: 5′-ATGTGAACCATGCAGTGTTGGTGG-3′
	Reverse: 5′-ATGCCGCAGGCGTTGTTCTTATTC-3′
*Tnfsf11*	Forward: 5′-TGTACTTTCGAGCGCAGATG-3′
	Reverse: 5′-CCCACAATGTGTTGCAGTTC-3′
*Nfatc1*	Forward: 5′-CGGCGCAAGTACAGTCTCAATGGC-3′
	Reverse: 5′-GGATGGTGTGGGTGAGTGGT-3′
*Acp5*	Forward: 5′-TCCTGGCTCAAAAAGCAGTT-3′
	Reverse: 5′-ACATAGCCCACACCGTTCTC-3′
*Tnfrsf11b*	Forward: 5′-GGCTGAGTGTTTTGGTGGACAG-3′
	Reverse: 5′-GCTGGAAGGTTTGCTCTTGTGA-3′
*Bglap*	Forward: 5′-GCAATAAGGTAGTGAACAGACT-3′
	Reverse: 5′-GTTTGTAGGCGGTCTTCAAGC-3′
*Alpl*	Forward: 5′-GGACAGGACACACACACACA-3′
	Reverse: 5′-CAAACAGGAGAGCCACTTCA-3′
*Axin1*	Forward: 5′-TGACTCTCCTTCCAGATCCCA-3′
	Reverse: 5′-TGCCCACACTAGGCTGACA-3′
*Ctnnb1*	Forward: 5′-CGCCTTTGCGGGAACAGGGTT-3′
	Reverse: 5′-ATGCGCACGCCCTCCACAAA-3′
*Ccn4*	Forward: 5′-ATCGCCCGAGGTACGCAATAG-3′
	Reverse: 5′-CAGCCCACCGTGCCATCAATG-3′
*Runx2*	Forward: 5′-AGGGACTATGGCGTCAAACA-3′
	Reverse: 5′-GGCTCACGTCGCTCATCTT-3′
*Gapdh*	Forward: 5′-ATCACTGCCACCCAGAAGAC-3′
	Reverse: 5′-GGATGCAGGGATGATGTTCT-3′

### Western blot analysis

Protein levels of SAA1 and G-CSF in sera were measured by western blot. Goat anti-mouse SAA1 antibody (Abcam) diluted at a ratio of 1:1000, Goat anti-G-CSF primary antibodies (Santa Cruz Biotechnology) diluted at a ratio of 1:2000 were used, followed by HRP-conjugated anti-goat IgG secondary antibodies (Santa Cruz Biotechnology). Immunoblots were visualized using the ECL Detection System (GE Healthcare).

### Micro-computed tomography (CT)

Intact tibia were dissected from mice, and muscle and soft tissues were removed. The bone was fixed in 10% formalin for 24–48 h and stored in 70% ethanol. A micro-CT scan was conducted using a Quantum GX2 micro-CT Imaging System (PerkinElmer, Waltham, MA, USA) using the following parameters: 90 kV, 88 μA, and with a voxel size of 50 μm.

### Histology

Tibia tissues were decalcified in 0.25 M EDTA (pH 7.5) and 4% paraformaldehyde solution for 4 weeks. The solution was refreshed every week. Paraffin-embedded tibia were sectioned and stained using a TRAP staining kit (Takara) according to manufacturer's instructions. This was followed by methyl green counterstaining.

### Fluorescence flow cytometry analysis

A single-cell suspension was obtained from lymph node, blood, and bone marrow cells. Blood and bone marrow cells were used after removing red blood cells (RBCs), using RBC lysis buffer (*i.e.*, 155 mM ammonium chloride, 12 mM sodium bicarbonate, and 0.1 mM EDTA). For intracellular staining, single-cell suspensions were fixed with 4% paraformaldehyde solution and treated with permeabilization buffer (90% methanol). The following antibodies were used; fluorescein isothiocyanate (FITC)-conjugated anti-CD4 (RM4-5; Thermo Fisher Scientific), FITC-conjugated anti-γδTCR (UC7-13D5; Thermo Fisher Scientific), FITC-conjugated anti-hematopoietic Lineage Antibody Cocktail (17A2, RA3-6B2, M1/70, TER-119, RB6-8C5; Thermo Fisher Scientific), PerCP-eFluor 710-conjugated anti-CD90.2 (30-H12; Thermo Fisher Scientific), phycoerythrin (PE)-conjugated anti-Ly-6G (1A8-Ly6g; Thermo Fisher Scientific), PE- or PerCP-conjugated anti-IL-17A (eBio17B7, Thermo Fisher Scientific), PE-conjugated anti-mouse CD254 (TRANCE, RANKL, IK22/5; Biolegend), and APC-conjugated anti-mouse Ly-6C (HK1.4; Thermo Fisher Scientific). All samples were acquired and analyzed using a BD Accuri C6 flow cytometer (BD Biosciences).

### Statistical analysis

Data are expressed as the mean ± standard deviation from at least three independent experiments. Significant differences between groups were calculated using two-tailed Student's *t*-test. For all analyses, *p* < 0.05 was considered statistically significant.

## Data availability

All data are contained within the article.

## Supporting information

This article contains supporting information.

## Conflict of interest

The authors declare that they have no conflicts of interest with the contents of this article.
