# Supplementary material for: Overexpression of hepatic serum amyloid A1 in mice increases IL-17-producing innate immune cells and decreases bone density
Source: J Biol Chem. 2021 Mar 26;296:100595. doi: 10.1016/j.jbc.2021.100595 (PMC8086136; doi:10.1016/j.jbc.2021.100595)
Supplement: Supplementary file 1 — Figures S1 to S6 [file mmc1.docx]

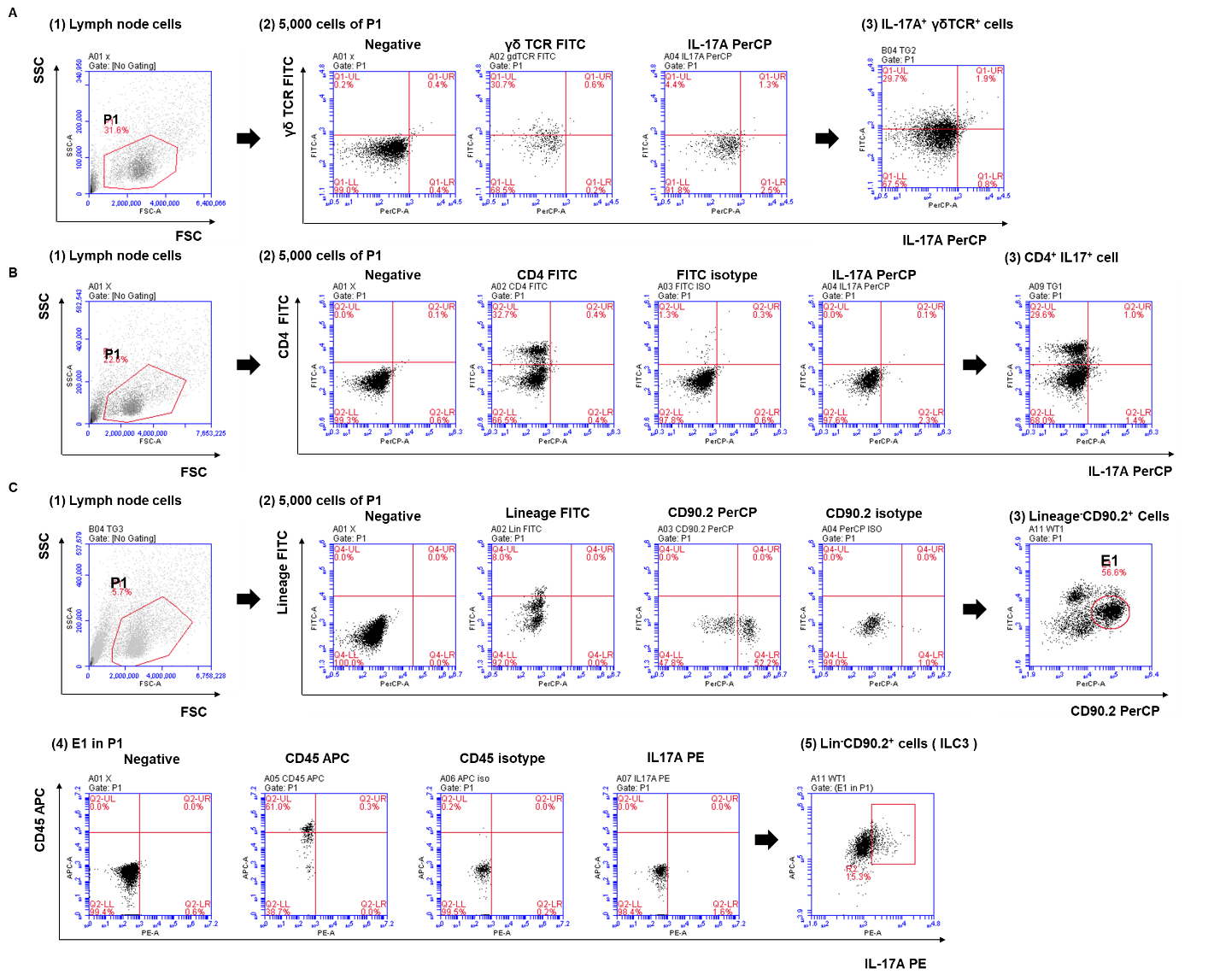


**Supplementary figure 1**. Gating strategy for γδTCR^+^ IL17A^+^ cells, Th17 and ILC3 cells located from lymph node cells. (1 of A, B, C) lymph node cells were gated to P1 through FSC and SSC. Cell populations corresponding to (A2) γδTCR^+^ IL17A^+^ cells, (B2) CD4^+^IL-17A^+^ cells and (C2) lineage^-^CD90.2^+^ cells were gated at 5,000 of P1 cells. (A3) γδTCR^+^ IL17A^+^ gated cells represent IL-17A secreting γδT cells. (B3) CD4^+^IL-17A^+^ cells indicate Th17 cells. (C3) Lineage^-^CD90.2^+^ gated cells were indicated as E1 cells. (C4) E1 cells were gated to CD45^+^ and IL-17A^+^ cell populations. (C5) Lineage^-^CD90.2^+^ CD45^+^ IL-17A^+^ gated cells represent ILC3 cells.


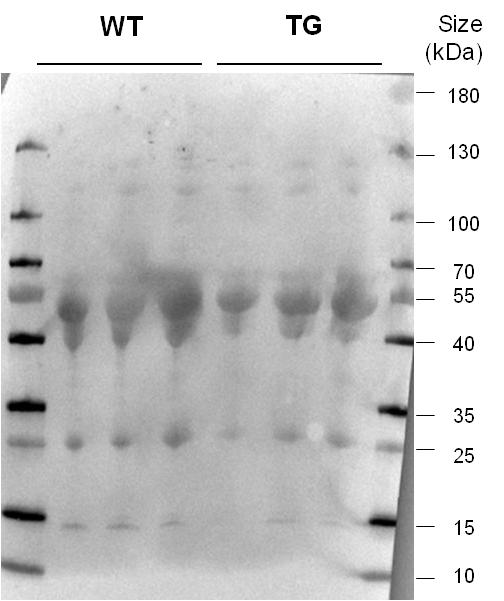


**Supplementary figure 2.** Ponceau S staining of western blot used in Figures 2A and 3A. The size of SAA1 is 12kDa, and the size of G-CSF is 19kDa.


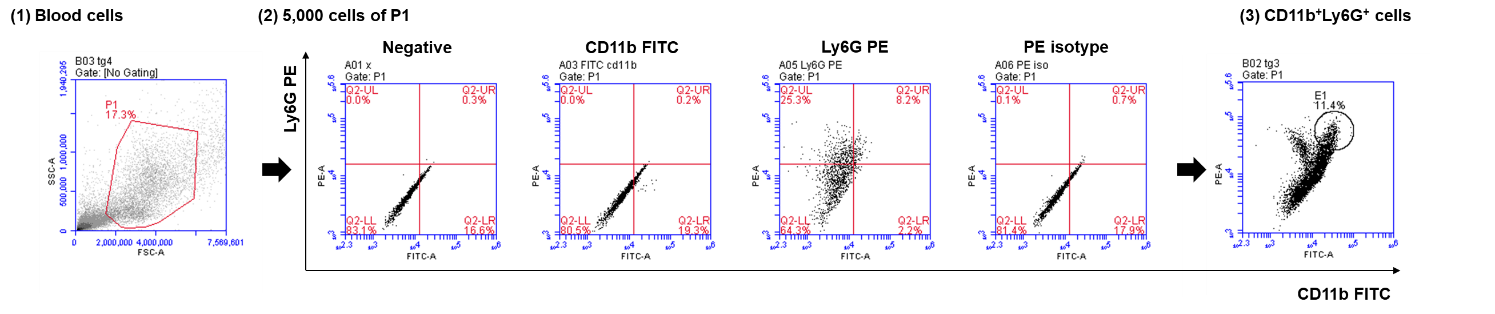
**Supplementary figure 3.** Gating strategy used to identify CD11b^+^Ly6G^+^ cells from blood. (1) Blood cells were gated to P1 cells through FSC and SSC. (2) Cell populations corresponding to CD11b^+^ and Ly6G^+^ were gated at 5,000 of P1 cells. (3) CD11b^+^ Ly6G^+^ gated cells represent neutrophils.


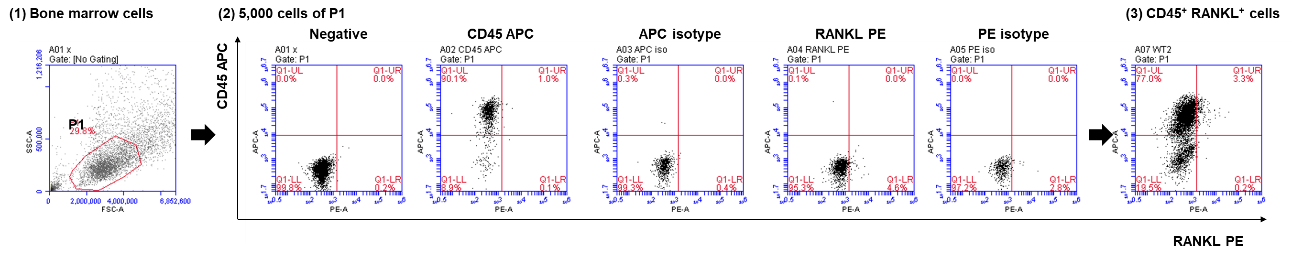


**Supplementary figure 4.** Gating strategy used to identify CD45^+^RANKL^+^ cells from bone marrow. (1) Bone marrow cells were gated to P1 cells through FSC and SSC. (2) Cell populations corresponding to CD45^+^ and RANKL^+^ cells were gated at 5,000 of P1 cells. (3) CD45^+^ RANKL^+^ gated cells are shown.


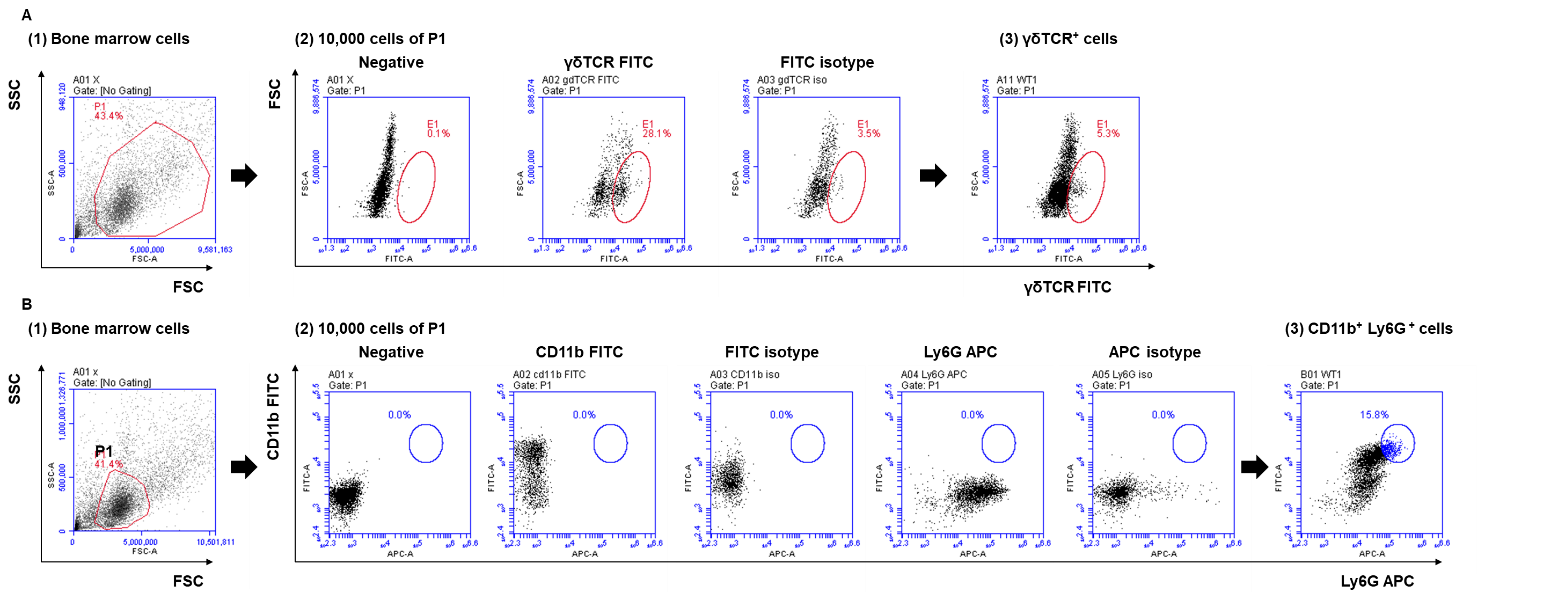


**Supplementary figure 5**. Gating strategy for γδTCR^+^ and CD11b^+^Ly6G^+^ cells from bone marrow. (A and B 1) Bone marrow cells were gated to P1 through FSC and SSC. Cell populations corresponding to (A2) γδTCR^+^ and (B2) CD11b^+^Ly6G^+^ were gated at 10,000 of P1 cells. (A3) γδTCR^+^ gated cells represent γδT cells and (B3) CD11b^+^Ly6G^+^ cells represent neutrophils from bone marrow.


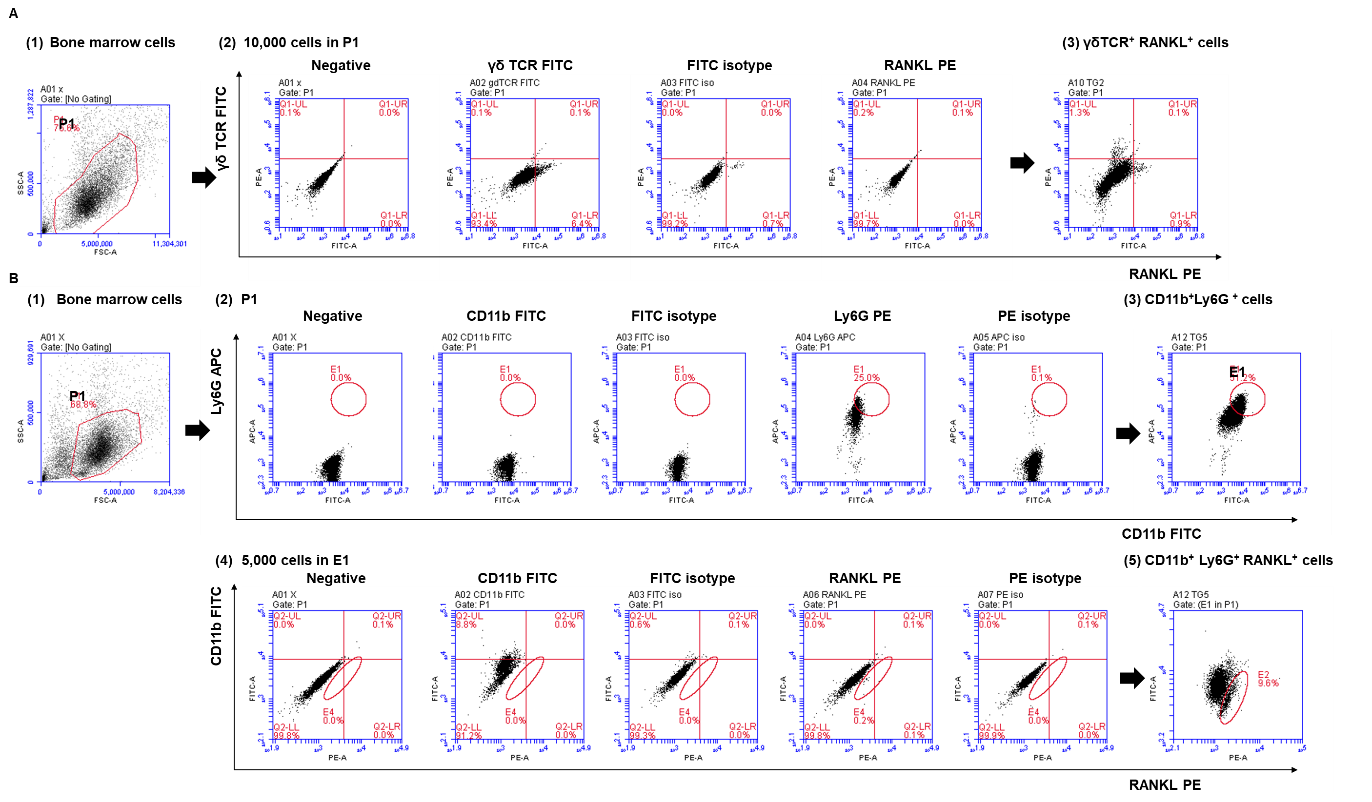


**Supplementary figure 6**. Gating strategy for γδTCR^+^RANKL^+^ and CD11b^+^Ly6G^+^RANKL^+^ cells from bone marrow. (A and B, 1) Bone marrow cells were gated to P1 through FSC and SSC. Cell populations corresponding to (A2) γδTCR^+^RANKL^+^ and (B2) CD11b^+^Ly6G^+^RANKL^+^ were gated to P1 cells. (A3) γδTCR^+^RANKL^+^ gated cells represent RANKL-secreting γδT cells. (B3) CD11b^+^Ly6G^+^ cells indicate E1 cells. (B4) 5,000 E1 cells were gated as CD11b^+^RANKL^+^ cell populations. (B5) CD11b^+^Ly6G^+^RANKL^+^ gated cells represent RANKL-secreting neutrophils from bone marrow.
